# Clinical Potential of microRNA-7 in Cancer

**DOI:** 10.3390/jcm4091668

**Published:** 2015-08-25

**Authors:** Jessica L. Horsham, Felicity C. Kalinowski, Michael R. Epis, Clarissa Ganda, Rikki A. M. Brown, Peter J. Leedman

**Affiliations:** 1 Laboratory for Cancer Medicine, Harry Perkins Institute of Medical Research, The University of Western Australia Centre for Medical Research, Perth, WA 6000, Australia; E-Mails: jessica.horsham@perkins.uwa.edu.au (J.L.H.); felicity.kalinowski@perkins.uwa.edu.au (F.C.K.); michael.epis@perkins.uwa.edu.au (M.R.E.); clarissa.ganda@perkins.uwa.edu.au (C.G.); rikki.brown@perkins.uwa.edu.au (R.A.M.B.); 2 School of Medicine and Pharmacology, University of Western Australia, Nedlands, WA 6009, Australia

**Keywords:** microRNA-7, microRNA replacement therapy, biomarker, cancer, tumour suppressor

## Abstract

microRNAs (miRNAs) are a family of short, non-coding RNA molecules that drive a complex network of post-transcriptional gene regulation by enhancing target mRNA decay and/or inhibiting protein synthesis from mRNA transcripts. They regulate genes involved in key aspects of normal cell growth, development and the maintenance of body homeostasis and have been closely linked to the development and progression of human disease, in particular cancer. Over recent years there has been much interest regarding their potential as biomarkers and as therapeutic agents or targets. microRNA-7 (miR-7) is a 23 nucleotide (nt) miRNA known primarily to act as a tumour suppressor. miR-7 directly inhibits a number of oncogenic targets and impedes various aspects of cancer progression *in vitro* and *in vivo*, however, some studies have also implicated miR-7 in oncogenic roles. This review summarises the role of miR-7 in cancer, its potential in miRNA-based replacement therapy and its capacity as both a diagnostic and prognostic biomarker.

## 1. Introduction

microRNAs (miRNAs) are a class of short (~22 nt), non-coding RNA molecules which play a central role, together with the RNA-induced silencing complex (RISC), in sequence specific post-transcriptional gene attenuation. miRNAs are generally evolutionarily conserved and their endogenous expression is tightly regulated [1]. Genes under the post-transcriptional control of miRNAs are manifold and consequently, miRNAs modulate the expression of proteins involved in various pathways essential for cell function, proliferation, differentiation, survival and development. The link between deregulated miRNA expression and cancer development and progression has been firmly established. Depending on their mRNA targets, miRNAs may act as oncogenes (oncomiRs) or tumour suppressors. microRNA-7 (miR-7) is considered to be a tumour suppressor miRNA in a number of malignancies such as breast [2], brain [3], head and neck [4], liver [5], colon [6] and melanoma [7]. However, there is also evidence to the contrary with a number conflicting reports suggesting both a tumour suppressive and oncogenic role for miR-7, particularly in lung cancers [8,9,10,11]. This review is focused on miR-7 and its clinical potential in cancer, as a therapeutic molecule in itself or as a target for overexpression. In addition we examine its potential as a prognostic and diagnostic biomarker.

## 2. microRNA-7 Expression and Regulation

### 2.1. Biogenesis

Expression of miR-7 stems from three loci in humans, *MIR7-1*, *MIR7-2* and *MIR7-3*. *MIR7-1* is located in the last intron of the widely expressed heterogeneous nuclear ribonucleoprotein K (*hnRNPK*) gene on chromosome 9 and is believed to be the most highly expressed source of mature miR-7 [12]. *MIR7-2* is found in an intergenic region of chromosome 15, and *MIR7-3* is located intronically within the pituitary gland specific factor 1 (*PGSF1*) gene on chromosome 19 [13]. Each miR-7 gene gives rise to three unique primary miRNA transcripts termed pri-miR-7-1, pri-miR-7-2 and pri-miR-7-3. Primary miRNA transcripts are commonly >1000 nt in length and contain stem-loop structures [1]. They are subsequently cleaved by Drosha to generate hairpin precursor miRNAs termed pre-miR-7-1, pre-miR-7-2 and pre-miR-7-3. Following Drosha cleavage, the resulting precursor miRNAs which are ~110 nt in length are transported to the cytoplasm where the terminal loop is removed by Dicer, creating a short duplex mature miRNA consisting of a miR-7-5p and miR-7-3p strand. To date, the majority of studies have concentrated on miR-7-5p which is commonly referred to simply as “miR-7”. One strand, termed the “guide strand” or “leading strand” becomes associated with RISC. The guide strand may be either the -5p or the -3p strand and is determined in part by the relative stability of the 5′ end and excess of purine *versus* pyrimidine composition [14]. The passenger strand, referred to as miRNA*, is considered inactive and is typically degraded. The miRNA subsequently guides RISC to target mRNA via sequence-specific recognition, providing an interface for interaction with the corresponding mRNA. Binding typically occurs at the 3′ untranslated region (3′-UTR) of mRNA transcripts, although examples exist of binding sites within the 5′ untranslated region (5′-UTR) or mapped coding regions. Complementarity is often imperfect and central bulging results in translational repression of the mRNA, however, in the event of complete complementarity, mRNA cleavage ensues with accelerated mRNA decay. Target site recognition is dependent on perfect base pairing at nucleotides 2–8 of the miRNA known as the “seed” region. miRNAs generally exert only modest repression on their targets and so their action is more akin to “fine-tuning” gene expression [15,16]. An in-depth discussion of miRNA biogenesis can be found in a recent review by Ha and Kim (2014) [1].

All three miR-7 loci give rise to the same mature miR-7 sequence which is evolutionarily conserved. However, it should be noted that alternative sequences of miRNAs termed isomiRs have been identified in RNA-seq studies and may have biological significance. These isomiRs potentially arise from AGO2 cleavage independent of Dicer, producing base substitutions and size variations and are thought to be functionally relevant, possibly cooperating with canonical miRNAs to target common molecules and pathways [17]. Although miR-7 is expressed widely at low levels, it is enriched in various regions of the brain, particularly the pituitary [18] (noting the location of *MIR7-3* in the intron of pituitary-specific *PGSF1*), hypothalamus [19] and pancreatic islets [20,21]. Studies suggest miR-7 may have a key role in pancreatic beta cell development and maturation and accordingly is postulated to be a therapeutic target in diabetes [22,23]. The complete role of miR-7 in the brain is yet to be fully elucidated, however recent studies suggest it has roles in brain and neuronal cell development [24]. The lack of miR-7 expression in non-neuronal tissues, despite the widespread expression of the miR-7 host gene *hnRNPK*, is thought to be governed at the processing rather than at the transcriptional level [25]. Expression of intronic miRNAs may also stem from their own promoter regions [26], as has been shown for *MIR7-1* [2,8].

### 2.2. Transcriptional and Post-Transcriptional Regulation

**Figure 1:**
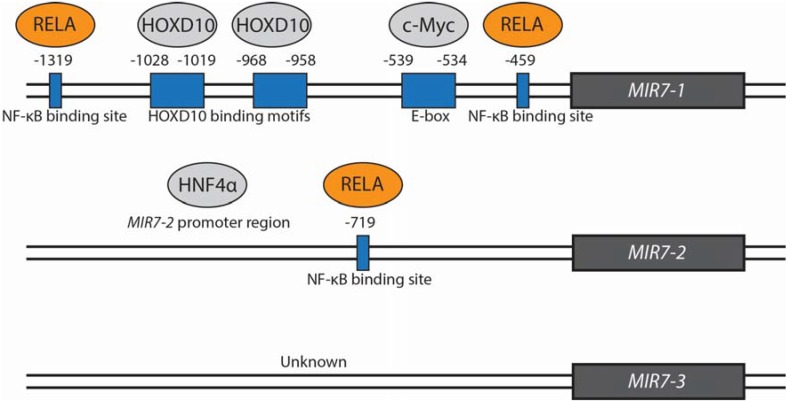
Transcriptional regulation of miR-7 by proteins confirmed to bind to *MIR7* promoter regions. Those shown in grey positively regulate miR-7 expression while those shown in orange negatively regulate miR-7 expression. The transcription factors HOXD10 and c-Myc bind to and stimulate expression from the *MIR7-1* promoter. HOXD10 may bind to two binding motifs −1019 to −1028 bp and −958 to −968 bp upstream of the *MIR7-1* transcription initiation site [2]. C-Myc has been found to bind to an E-box motif at positions −534 to −539 bp upstream of *MIR7-1* [8]. HNF4α similarly binds and stimulates expression from the *MIR7-2* promotor region. The exact location is not described [5]. RELA binds to three predicted NF-κB binding sites at −459 and −1391 bp in the *MIR7-1* and −719 bp *MIR7-2* promoters [31]. Proteins which bind and stimulate or inhibit expression from a *MIR7*-3 promoter are currently unknown.

The regulation of mature miR-7 expression occurs at the transcriptional level as well as at various stages throughout the miRNA maturation process, and there are many examples. At the transcriptional level, miR-7 expression has been shown to be promoted by epidermal growth factor receptor (EGFR) signaling in lung cancer via Rat sarcoma (Ras)/extracellular signal-regulated kinase (ERK)/v-Myc avian myelocytomatosis viral oncogene homolog (c-Myc) and phosphoinositide 3-kinase (PI3K)/v-Akt murine thymoma viral oncogene homolog (Akt) pathways. Whilst the exact mechanism of miR-7 stimulation via the PI3K/Akt pathway is yet to be identified, the transcription factor c-Myc was found to directly bind and stimulate expression from the *MIR7-1* promoter [8]. This finding is supported by an earlier study which also found miR-7 upregulation as a result of c-Myc expression in lymphoma [27]. Other transcription factors have similarly been involved in promoting miR-7 expression via directly interacting with the promoter regions of miR-7 genes including homeobox D10 (HOXD10) via the *MIR7-1* promoter region in breast cancer [2] and Hepatocyte Nuclear Factor 4 alpha (HNF4α) via the *MIR7-2* promoter in hepatocellular carcinoma (HCC). HNF4α was identified as part of a feedback loop also involving miR-124, miR-21 and nuclear factor-kappa B (NF-κB) [5]. Binding of these proteins to *MIR7-1* and *MIR7-2* promotor regions is illustrated in Figure 1. The transcription factor Forkhead box P3 (FOXP3) which also positively regulates miR-7 expression in breast cancer [28] has been found to have potential binding regions in the locality of *MIR7-1* and *MIR7-2* genes [29]. miR-7 expression is further promoted by hepatitis B virus X protein (HBx) in hepatitis B virus-associated HCC. The transduction of signals between HBx and miR-7 activation is postulated to involve nuclear I kappa B kinase alpha (IKKα) and I kappa B kinase (IKK)/NF-κB signaling pathways, however, this relationship is yet to be elucidated [30]. A recent study in gastric cancer found miR-7 to be involved in a negative feeback loop with IKKε and v-Rel avian reticuloendotheliosis viral oncogene homolog A (RELA). miR-7 targets and inhibits IKKε and RELA expression, and IKKε and RELA were found to suppress pri-miR-7 expression. Direct binding of RELA to both *MIR7-1* and *MIR7-2* promoter regions was confirmed [31]. Further, ubiquitin-specific peptidase 18 (Usp18) negatively regulates miR-7 expression. Knockdown of Usp18 was found to increase expression of miR-7 host genes and intergenic pri-miR-7-2 and subsequently mature miR-7 [32]. miR-7 expression is further negatively regulated by the oncogenic long non-coding RNA, Hox transcriptase antisense RNA (HOTAIR). HOTAIR indirectly inhibits miR-7 expression via HOXD10 suppression. Downregulated HOTAIR showed an anti-correlative relationship with both HOXD10 and miR-7 in MDA-MB-231 breast cancer cells and miR-7 was inversely correlated with HOTAIR expression in breast cancer patients [33].

Post-transcriptional regulation of miR-7 is promoted by serine/arginine-rich splicing factor 1 (SRSF1, also known as SF2/ASF) in a splicing-independent fashion. SRSF1 promotes maturation of many miRNAs including miR-7 via enhancing Drosha cleavage of the primary transcript. miR-7 in turn targets and inhibits translation of SRSF1 via its 3′UTR, completing a negative feedback loop [34]. Conversely, the RNA binding protein, Human antigen R (HuR), negatively affects miR-7 maturation. Lebedeva *et al.* (2011) showed HuR knockdown to be negatively correlated to the specific and substantial upregulation of miR-7 [35]. Li *et al.* (2013) similarly found miR-7 expression to be impeded by toll like receptor 9 (TLR9)-induced HuR upregulation in lung cancer cells [36]. Furthermore, Musashi homolog 2 (MSI2) was found to bind to the terminal loop of the pri-miR-7 transcript in an HuR-dependent manner in non-neural cells resulting in failure of the pri-miR-7-1 transcript to mature [25]. Quaking homologs, KH domain RNA binding 5 and 6 (QKI-5 and QKI-6), have also been implicated in the failure of miR-7-1 to be processed into mature miR-7 and exported to the cytoplasm in glioblastoma. QKI binding sites were found in pri-miR-7-1 and pri-miR-7-2 but not pri-miR-7-3. QKI-5 and QKI-6 are speculated to increase association of miR-7-1 with Drosha [37].

A circular RNA (circRNA) sponge for miR-7 termed “ciRS-7” (also referred to as CDR1NAT, CDR1-AS and CDR1as) has been recently identified [38,39]. ciRS-7 is derived from the antisense transcript of the coding CDR1 gene [38] and is highly and stably expressed in human and mouse brain [38]. ciRS-7 is suggested to act as a competing endogenous RNA or miRNA “sponge” in neuronal tissues and contains >70 seed-matched miR-7 binding sites. The pattern of ciRS-7 expression in the mouse brain closely aligns with that of miR-7, especially in the hippocampus and neocortex [39] and in the developing brain of mouse embryos [40]. Whilst ciRS-7 is able to considerably attenuate miR-7 activity and thereby reduce repression of miR-7 targets [39], the biological function of ciRS-7 is yet to be defined. It is suggested that ciRS-7 may act as a buffer of miR-7 activity by competing with miR-7 targets, thereby reducing the availability of miR-7 for low-affinity target mRNAs. To add an additional level of regulation, miR-671 via near-perfect complementarity has been shown to cause RISC-induced endonucleolytic ciRS-7 degradation [38]. It is speculated that upon ciRS-7 degradation, sequestered miR-7 is released. Therefore, miR-671 could possibly be considered a positive regulator of miR-7 either by release of ciRS-7 bound miR-7 or by reducing the number of available ciRS-7 molecules for miR-7 sequestration [12]. In summary, circRNAs that act as miRNA sponges are only beginning to be understood and their role in cellular homeostasis is yet to be elucidated. A summary of molecules involved in miR-7 regulation can be found in Table 1.

**Table 1:** Summary of miR-7 regulatory molecules and their effect on miR-7 expression in cancer cell lines.

Regulatory Molecule/Pathway	miR-7 Up- (↑)/Down- (↓) Regulation	Action	Direct/Indirect Interaction	Cancer Type	Reference
EGFR signaling	↑	Via Ras/ERK/Myc and additionally by PI3K/Akt pathways	Indirect	Lung	Chou *et al.* (2010) [8]
c-Myc	↑	Binds and stimulates expression from the *MIR7-1* promoter	Direct	Lung	Chou *et al.* (2010) [8]
HOXD10	↑	Binds and stimulates expression from the *MIR7-1* promoter	Direct	Breast	Reddy *et al.* (2008) [2]
HNF4α	↑	Interacts with *MIR7-2* promoter	Direct	Liver	Ning *et al.* (2014) [5]
FOXP3	↑	Predicted binding regions in proximity to *MIR7-1* and *MIR7-2* loci	Not confirmed	Breast	McInnes *et al.* (2012) [28]
HBx	↑	Postulated to involve IKKα and IKK/NF-κB signaling	Indirect	Liver	Chen *et al.* (2013) [30]
RELA	↓	Binds to *MIR7-1* and *MIR7-2* promoter regions	Direct	Gastric	Zhao *et al.* (2015) [31]
Usp18	↓	Mechanism not identified	Not confirmed	Cervical, Head and neck, Brain	Duex *et al.* (2011) [32]
HOTAIR	↓	Via inhibiting HOXD10	Indirect	Breast	Zhang *et al.* (2014) [33]
SF2/ASF	↑	Binds to pri-miR-7 and promotes maturation via enhancing Drosha cleavage	Direct	Cervical	Wu *et al.* (2010) [34]
HuR	↓	Hypothesised to represses miR-7-1 processing which may involve HuR binding in the intron of *hnRNPK* which hosts the *MIR7-1* gene	Not confirmed	Cervical, Lung	Lebedeva *et al.* (2011) [35], Li *et al.* (2013) [36]
TLR9 signaling	↓	Via HuR upregulation which is suggested to involve the PI3K/Akt pathway	Indirect	Lung	Li *et al.* (2013) [36]
MSI2	↓	Binds to the terminal loop of the pri-miR-7 transcript in an HuR-dependent manner resulting in failure of the pri-miR-7-1 transcript to mature	Direct	Cervical, Brain	Choudhury *et al.* (2013) [25]
QKI 5 and QKI 6	↓	Bind to QKI response elements in pri-miR-7-1 resulting in processing failure (binding sites also identified in pri-miR-7-2)	Direct	Brain	Wang *et al.* 2013 [37]
ciRS-7	↓	Contains >70 seed-matched miR-7 binding sites that can sequester miR-7	Direct	Proof of concept demonstrated in HeLa and HEK293 cells	Hansen *et al.* 2013 [39], Memczak *et al.* 2013 [40]

## 3. The Role of microRNA-7 in Cancer

### 3.1. miR-7 is a Tumour Suppressor

Key molecular targets of miR-7 in various tumourigenic processes and pathways have been systematically and extensively reviewed recently by Kalinowski *et al.* (2014) and by Gu *et al.* (2015) [41,42]. Expression profiling data from our own group and others suggests that miR-7 targets ~100–200 mRNAs in cancer cells, many of those targets containing putative miR-7 binding sites, so that there is significant enrichment of miR-7 activity [4,43]. One of the additional remarkable features of miRNAs such as miR-7, is that they have the potential to target multiple parts of a signaling pathway simultaneously (e.g., EGFR) which can produce a more profound inhibition of signaling compared to targeting a single site of the pathway, with a tyrosine kinase inhibitor, such as erlotinib.

The significance of miR-7 in cancer is well-documented having been shown to directly target and inhibit key oncogenic signaling molecules involved in cell cycle, proliferation, invasion and metastasis. For example, Proteasome Activator Subunit 3 (PA28γ) which promotes cell cycle progression has been shown to be directly targeted by miR-7 in the hamster ovarian cell line CHO, non-small cell lung cancer (NSCLC) and breast cancer via its 3′-UTR [44,45,46]. Shi *et al.* (2015) reported that miR-7 suppresses cell proliferation and induces G0/G1 phase arrest and apoptosis in breast cancer in part, via its interaction with PA28γ [46]. Moreover, miR-7 was also shown to cause cell cycle arrest in G1 phase by directly targeting cyclin E1 (CCNE1) in HCC [47].

miR-7 has also been shown to inhibit proliferation *in vitro* and importantly, tumour growth *in vivo*, with regulation of EGFR commonly being attributed to this effect [4,48,49]. EGFR is a well described target of miR-7, is a prominent regulator of normal cell differentiation, development and proliferation, and is commonly targeted for therapy in cancer [3,10,32,43,50,51,52]. Additionally, miR-7 affects the activity of multiple oncogenic molecules in the EGFR signaling cascade such as Akt and ERK1/2 [4,53], V-Raf-1 murine leukemia viral oncogene homolog (RAF1) [4,10,43,53], P21 protein (Cdc42/Rac)-activated kinase 1 (PAK1) [2,51], activated CDC42 kinase 1 (ACK1) [51], phosphatidylinositol-4,5-bisphosphate 3-kinase, catalytic subunit delta (PIK3CD), mammalian target of rapamycin (mTOR), phosphoprotein 70 ribosomal protein S6 kinase (p70S6K) [54] and PI3K [53] across several cancer types, demonstrating broad regulatory control over this signaling network.

miR-7 also targets key regulators of migration, invasion and epithelial-mesenchymal transition (EMT). Molecules such as focal adhesion kinase (FAK) [55,56], kruppel-like factor 4 (KLF4) [57], insulin-like growth factor-1 receptor (IGF1R) [58], insulin receptor substrate 1 (IRS-1) [2], insulin receptor substrate 2 (IRS-2) [7] and SET domain bifurcated 1 (SETDB1) [33] are all attributed to these processes. An example of this is SETDB1, which is involved in maintaining stem cell state, and is downregulated by miR-7 leading to partial reversal of EMT and inhibition of invasion and metastasis in breast cancer stem cells isolated from the MDA-MB-231 cell line. This effect can be explained by reduced activation of Signal transducer and activator of transcription 3 (STAT3) as a result of SETDB1 downregulation. SETDB1 was found to bind directly to the promoter of STAT3 and induce its expression. In contrast, knockdown of SETDB1 using RNA interference resulted in decreased STAT3 expression and activation [33]. Similarly, in an earlier study, Wang *et al.* (2013) showed that miR-7 transfected into glioma cells reduced the active phosphorylated form of STAT3 [59]. In addition, Ning *et al.* (2014) have reported miR-7 can inhibit metastasis in HCC through perturbation of NF-κB signaling by way of directly targeting and decreasing RELA and subsequently NF-κB activation [5]. Other *in vivo* studies have reported miR-7 to inhibit angiogenesis in glioblastoma xenografts [60], suppress tumour progression in gastric cancer [61] and play a role in the de-repression of epigenetically silenced tumour suppressor genes, which result in decreased colony formation and cell cycle progression in breast cancer [62].

### 3.2. miR-7: The Oncogene?

Whilst miR-7 expression has frequently been reported to be downregulated in several malignancies [6,7,10,33,41,43,45,54,58], increased levels have been associated with tumour aggressiveness, most notably in oestrogen receptor positive/lymph node negative (ER+/LNN) breast cancer [63], urothelial carcinoma [64] and in Human papillomavirus (HPV) infected cervical cancer patients [65]. Additionally, viral oncogene E6/E7 expression in the HPV-positive HeLa cell line was associated with upregulated miR-7 [66]. In colorectal cancer (CRC), miR-7 was found to be upregulated in advanced cancers and in selected cell lines (SW480, DLD-1, and COLO201) compared to normal mucosa. In addition, transfection with anti-miR-7 was shown to suppress cell growth in DLD-1 and COLO201 [67]. miR-7 was also reported to be increased in the stool of CRC patients, giving rise to the notion of a screening method for CRC [68]. In contrast to these examples, many reports suggest a tumour suppressive role for miR-7 in CRC. Zhang *et al.* (2013) reported miR-7 to be downregulated in CRC tumours and in six out of seven CRC cell lines when compared to normal colon tissue (these cell lines included SW480 and DLD-1) [6]. In addition, Suto *et al.* (2015) found low miR-7 expression to be associated with poor prognosis in CRC and showed miR-7 could inhibit proliferation in SW480 cells [48]. Zhang *et al.* (2013) found miR-7 overexpression resulted in reduced proliferation and induced G1 phase arrest and apoptosis via targeting yin yang 1 transcription factor (YY1) in CRC [6] and Xu *et al.* (2014) showed miR-7 targets the protein X-ray repair complementing defective repair in Chinese hamster cells 2 (XRCC2) to inhibit proliferation and induce apoptosis [69].

Conflicting reports have also emerged regarding the role of miR-7 in lung cancer. Chou *et al.* (2010) reported miR-7 to be induced via EGFR/Ras/ERK/Myc signaling and subsequently promote cell proliferation and tumour formation. However, miR-7 overexpression was also shown to attenuate EGFR expression in lung adenocarcinoma CLI-5 cells [8], suggesting the existence of an EGFR/miR-7 regulatory loop. Studies carried out in the epithelial NSCLC cell line A549 have demonstrated varied roles for miR-7. The findings of Chou *et al.* (2010) are supported by an earlier study which found that inhibiting miR-7 downregulated A549 cell growth [70]. Meza-sosa *et al.* (2014) showed that miR-7 induced proliferation and migration in A549 cells stably overexpressing miR-7, suggesting miR-7 may act as an oncomiR in an epithelial context. To strengthen this argument, naturally immortalised skin cells HaCaT also exhibited enhanced proliferation upon stable miR-7 overexpression. This was found to be due to direct downregulation of KLF4, a transcription factor which mediates diverse cellular processes including proliferation, by miR-7 [9]. In contrast, Rai *et al.* (2011) overexpressed miR-7 episomally and reported no significant growth inhibition in A549 cells, but showed suppressed growth in EGFR-addicted cell lines such as the NSCLC cell lines PC-9, H3255 and H1975. They did however observe much higher miR-7 levels in EGFR-addicted cells compared to non-addicted cells, suggesting an EGFR-mediated activation of miR-7 consistent with the findings of Chou *et al.* (2010) [10]. In work by Xiong *et al.* (2011), transient miR-7 overexpression inhibited migration, proliferation and induced apoptosis in A549 cells through targeting the anti-apoptotic molecule B-cell lymphoma 2 (BCL-2) [11]. We have found miR-7 to inhibit EGFR expression and signaling in A549 cells, consistent with it having a tumour suppressive effect [43]. In summary, clearly the role of miR-7 in lung cancer is more complex than initially envisaged, and may be particularly cell type specific and possibly dependent on the method of influencing miR-7 expression experimentally.

### 3.3. Genetic Influence on the Role of miR-7

The regulatory capacity of miR-7 is complex, given the numerous targets reported across many cell types. KLF4, a known target of miR-7 [9,57], elicits context-dependent oncogenic and tumour suppressive responses [71] and indeed, oncogenesis has been reported as a result of KLF4 suppression by miR-7 [9], as well as the opposite [57]. Similarly, with respect to the mutational profile of the cell, STAT3 (an indirect target of miR-7) can either promote or suppress tumourigenesis depending on biochemical and genetic factors [72,73]. Hence, the role/s of miR-7 may be adversely affected by the cells mutational background. Rai *et al.* (2011) suggest that the level of EGFR-addiction will play an important role in the effect of miR-7 [10]. Also, as observed in the studies conducted in A549 cells mentioned above [9,10,11,43,70] the experimental approach could be responsible for conflicting observations [41], which include scenarios whereby miR-7 is over- or under- expressed, the degree of miR-7 overexpression within the cell or whether miR-7 overexpression is sustained.

Given miR-7 is demonstrated to participate in feedback and “feedforward” loops, as well as regulating several transcription factors, changes in miR-7 expression may result in a “ripple” effect; that is, the indirect regulation of the expression of other genes, and even miRNAs. To emphasise this point, a study investigating miR-7 transient overexpression in ovarian cancer cells reported a change in the expression of hundreds of genes in diverse pathways; however, only ~20% of the regulated genes were predicted to be direct targets, concluding that the majority of the observed changes to gene expression are an indirect consequence of miR-7 expression and effect [74].

## 4. microRNA-7 Has Biomarker Potential

miRNAs have great potential as predictive, diagnostic and prognostic biomarkers both for cancer and other diseases, such as schizophrenia [75]. Reports indicate that free circulating miRNAs stably exist in body fluids such as blood serum, saliva [76] and urine [77]. It is hypothesised that these miRNAs have been secreted by cells in exosomes allowing for their inherent stability and resistance to RNase activity, which would otherwise degrade exogenous sources of miRNA [78,79]. Exosome secreted miRNAs found in blood and other body fluids are thought to act in cell-to-cell communication [79]. Microvesicle-free miRNA in body fluids may also exist stably associated with argonaute RISC catalytic component 2 (AGO2) [80] or high-density lipoprotein (HDL) [81]. These miRNAs provide a readily accessible and minimally invasive source for biomarker testing. miRNAs identified as potential biomarkers for cancers have also been measured in urine [82,83,84], saliva [85], and stool [86,87] and can be found in most body fluids [88]. Alternatively, miRNA expression may also be profiled directly from tumours and tissues or from circulating tumour cells (CTCs). CTCs represent the most preferable option as they offer a more reliable representation of the tumour miRNA profile than cell-free miRNAs and can be isolated relatively non-invasively, however, their isolation from the leukocyte background is currently challenging [89,90]. Biomarker miRNAs may not only be useful in diagnosis, especially for asymptomatic cancers such as pancreatic cancer which have no early detectable signs/symptoms, but particularly in patient stratification and even for identifying tumour origin from secondary lesions based on similarities in miRNA signatures [91].

In a study conducted by Wang *et al.* (2015), miR-7 was identified as one of three miRNAs (along with miR-93 and miR-409-3p) from an array of 723 human miRNAs, which were found to be powerful predictors of CRC. This panel of miRNAs could be used to distinguish CRC patients from healthy patients, as well as early stage CRC (nonmetastatic) and late stage CRC (metastatic) from healthy patients with great accuracy. The miRNAs were isolated from blood plasma, potentially preventing healthy patients from having to undergo uncomfortable and unnecessary colonoscopies [92]. A small proof-of-concept study by Ahmed *et al.* (2013), also conducted in CRC, found miR-7, among eleven other miRNAs, to be increased in stool samples from a small cohort of CRC patients when compared with healthy controls. This finding highlights the availability of miRNAs from stool samples which may be useful biomarkers for CRC [68]. Kitano *et al.* (2012) found miR-7 to be a useful biomarker for the prediction of benign thyroid tumours from malignant thyroid cancer, specifically in those cases where diagnosis is difficult to ascertain from fine-needle aspiration biopsies. The model was highly sensitive with a negative prediction value of 100%. Therefore, the model could correctly identify benign tumours, but lacked adequate positive prediction (identification of malignant lesions) [93]. This highlights the potential clinical usefulness of miRNA biomarkers and also the need for further investigation to achieve greater specificity and sensitivity in diagnostic assays.

In many cancer types, high or low levels of miR-7 have been associated with poor or more promising prognoses and may be harnessed for biomarker profiling. In a study identifying miRNA biomarkers involved in the progression of hormone-sensitive prostate cancer to castrate-resistant prostate cancer (CRPC), Santos *et al.* (2014) identified miR-7 levels in peripheral whole blood as a useful prognostic biomarker for CRPC development. Higher miR-7 levels in peripheral whole-blood in combination with high-Gleason score tumours was correlated with significantly earlier progression to castrate resistance and further trended toward lower overall survival of patients [94]. In contrast, in another cancer phenotype, Okuda *et al.* (2013) have suggested that low levels of miR-7 and inversely high KLF4 expression may be useful as prognostic biomarkers for predicting brain metastasis of breast cancer [57]. Thus, further evaluation of miR-7 expression in carefully selected clinical cohorts will be required to refine the potential application as a biomarker.

## 5. Potential for microRNA-7 in Cancer Therapy

### 5.1. miR-7 Replacement Therapy Alone and in Combination with Current Therapeutic Agents

miRNAs present themselves as attractive potential therapies, either in the context of replacement of tumour suppressors or suppression of oncomiR activity. miRNA therapy can be broadly assigned into two categories, replacement and inhibition. As the overwhelming majority of reports suggest miR-7 acts as a tumour suppressor, there is increasing focus on replacement therapy. One strategy is systemic administration and delivery of miR-7. Two methods have been used to successfully deliver miR-7 *in vivo* to treat cancer. In a study developed by Babae *et al.* (2014), a miR-7 mimic was systemically delivered using clinically viable, biodegradable, targeted polyamide nanoparticles. This achieved successful inhibition of tumour growth and vascularisation in a glioblastoma xenograft [60]. In an earlier study, Wang *et al.* (2013) was able to inhibit glioma xenograft growth and metastasis using a plasmid based miR-7 vector systemically delivered by encapsulation in a cationic liposome formulation [59].

miRNA-based replacement therapy is most likely to be given as a tumour suppressive miRNA in combination with other therapeutic agents, such as tyrosine kinase inhibitors. It has been suggested that miR-7 may enhance the effect of current therapeutic drugs. A number of studies have demonstrated restored therapeutic sensitivity to targeted treatments as a result of miR-7 expression *in vitro*. Results from our laboratory showed miR-7 was able to increase the sensitivity of erlotinib-resistant head and neck cancer cells to erlotinib [4]. An earlier study by Pogribny *et al.* (2010) reported miR-7 expression directly targeted and significantly inhibited multidrug resistance-associated protein 1 (MPR1) which increased sensitivity to cisplatin in cisplatin-resistant breast cancer [95]. An *in vitro* study by Suto *et al.* (2015) showed miR-7 overexpression increased sensitivity to cetuximab in HCT-116 and SW480 cetuximab-resistant CRC cells harbouring a Kirsten rat sarcoma viral oncogene homolog (KRAS) mutation. However, miR-7 was ineffective in the CRC cell line HT-29 which expresses a v‑Raf murine sarcoma viral oncogene homolog B (BRAF) mutation. This was reportedly due to miR-7 targeting of not only EGFR but also RAF-1 which plays an key role in mutant KRAS signaling, but not in BRAF mutants [48]. Additionally, miR-7 was found to increase sensitivity of NSCLC to paclitaxel (PTX) by promoting PTX-induced apoptosis [96]. Results from microarray and qPCR analyses in gefitinib resistant A549 cells compared to the parental A549 cell line found miR-7 to be downregulated which suggests possible involvement in the development of gefitinib resistance, however further study is required to identify whether miR-7 has the potential to improve gefitinib sensitivity [97]. Acquired resistance to chemotherapy is common in many patients and presents a real clinical challenge. miRNAs that increase the sensitivity of cancers to current therapies offer potential for use in combinational therapy. miRNAs as therapeutics have the added advantage of concurrently regulating multiple molecules and members of various pathways which may reduce the chance of acquired resistance developing such as often the case with inhibitors that target single molecules or pathways.

### 5.2. Potential for Small Molecule Activation of microRNA-7

As previously discussed, there is the potential to regulate miRNAs at both the transcriptional and processing level. High-throughput screens have identified compounds with demonstrated potential to both promote and inhibit miRNA transcription. One example of this is curcumin, which has been shown to upregulate a number of miRNAs, including miR-7, in pancreatic cancer [98]. Also, the antibacterial enoxacin was observed to increase the processing of certain miRNAs, including miR-7, from the precursor form to the mature form in RKO and HCT-116 CRC cell lines [99] while in another study, the histone deacetylase inhibitor Thichostatin A (TSA) was found to induce miR-7 in MDA-MB-231 breast cancer cells resulting in inhibition of EGFR expression [100]. Experimentally validated relationships between small molecules and miRNA expression in various species are compiled and accessible in the SM2miR database [101]. Whilst this highlights the potential for small molecule mediated miRNA regulation, it must be emphasised that the action of these small molecules is often nonspecific, which raises the possibility of significant “off target” effects.

## 6. Conclusions

Whilst the broad coordinated simultaneous downregulation of multiple gene networks with miRNAs is an attractive therapeutic option, the potential for off-target effects is still to be well defined and requires further investigation. Nonetheless, miRNA therapy may offer clinical practice the ability to treat diseases at a network level rather than targeting a single gene. In the interim, methods to achieve effective systemic administration of miRNAs are being actively pursued; however there are several hurdles to overcome before miRNA replacement therapy becomes routinely clinically achievable for diseases beyond the liver. Alternatively, several publications have highlighted the potential for small molecules to affect and regulate miR-7 expression, opening up further therapeutic possibilities. Whilst the topic of miR-7 in cancer is the subject of a small number of reports suggesting an oncomiR-phenotype, the vast majority of literature indicates miR-7 is a tumour suppressor with many prominent oncogenic targets. In addition, several studies have demonstrated the clinical potential of miR-7 as a biomarker in diagnosis and prognosis of disease. One of miR-7’s key clinical applications may relate to its capacity to sensitise tumours that are resistant to other targeted therapies (e.g., erlotinib). In summary, the accumulating *in vitro* and *in vivo* preclinical data continues to build a strong case for the use of miR-7 replacement therapy in specific cancers, especially HCC and head and neck cancer. It will be of great interest in the next few years to see if this prediction comes to fruition.
